# A semi-automatic toolbox for markerless effective semantic feature extraction

**DOI:** 10.1038/s41598-022-16014-8

**Published:** 2022-07-13

**Authors:** Vito Paolo Pastore, Matteo Moro, Francesca Odone

**Affiliations:** 1 Italian Institute of Technology (IIT), Genova, Italy; 2 Department of Informatics, Bioengineering, Robotics and Systems Engineering (DIBRIS), University of Genova and with the Machine Learning Genoa (MaLGa) Center, Genova, Italy

**Keywords:** Image processing, Machine learning, Software

## Abstract

VisionTool is an open-source python toolbox for semantic features extraction, capable to provide accurate features detectors for different applications, including motion analysis, markerless pose estimation, face recognition and biological cell tracking. VisionTool leverages transfer-learning with a large variety of deep neural networks allowing high-accuracy features detection with few training data. The toolbox offers a friendly graphical user interface, efficiently guiding the user through the entire process of features extraction. To facilitate broad usage and scientific community contribution, the code and a user guide are available at https://github.com/Malga-Vision/VisionTool.git.

## Introduction

Human motion understanding is a relevant task in many fields in science and medicine. Quantitative and qualitative motion analysis, e.g. predicting and describing human behavior while performing different actions, is essential in neuroscience to understand the brain behaviour in both physiological and pathological conditions^1–4^. Moreover, it is helpful for human-computer interaction applications, where a computer can be controlled with dedicated gestures^5–7^, for human-robot interaction, where a robot can detect change in human landmarks to provide dedicated assistance^8,9^ and for augmented reality applications for gaming and rehabilitation^10,11^. Lastly, human motion understanding is largely adopted in proxemic recognition in order to predict how people interact^12,13^. Nowadays, the gold standard techniques commonly adopted to characterize and study human motion rely on wearable sensors, motion capture systems and physical markers placed on the body skin^14^. However, markers are intrusive, they may limit natural movements, and their location is assigned a priori by expert operators, making the study of human motion biased^15^. Furthermore, they are cumbersome, making the analysis of motion patterns problematic in certain application fields^16,17^.

For these reasons, recently, RGB video analysis has become a possible alternative to marker-based systems to perform human motion analysis^18,19^. This is due to the increasing progress—in terms of accuracy and computational cost—of deep learning algorithms in solving computer vision problems^20^. In particular, recent advances on pose estimation algorithms based on deep neural networks are opening the possibility of adopting efficient methods for extracting motion information starting from common red–green–blue (RGB) video data^21^. Pose estimation consists in identifying the position of the subject body in images or image sequences, and it involves body landmark points detection and skeleton estimation. The latter may be carried out exploiting spatial^22–24^ or spatio-temporal relationships^25^.

Besides full body human pose estimation, in many application scenarios it is not necessary to retrieve people skeleton, but there is the need to focus and localize specific features in the image planes (as usually done for body keypoints detection in pose estimation algorithms). In fact, there is a large variety of application fields in science where the availability of accurate algorithm for the detection of semantic features in the image plane may be crucial, including the analysis of body parts and human faces, animal pose-estimation^24^, small objects localization^26^ or biological image analysis.

Having in mind this broad range of applications, it becomes clear that versatility is a fundamental feature for a toolbox aiming to provide general-purpose semantic features extraction. In particular, it requires: (1) the possibility to define the set of high-level features to detect (e.g., animal joints, the center of a cell body, a set of face descriptors, etc.); (2) no assumption on input data, which may be a video or a set of static and uncorrelated images; (3) high accuracy with minimal training data because obtaining annotated data is not a trivial process. In fact, annotation is time-consuming and user-dependent. Moreover, the availability of training data may be intrinsically limited in certain application fields (e.g., biology and medicine).

**Figure 1:**
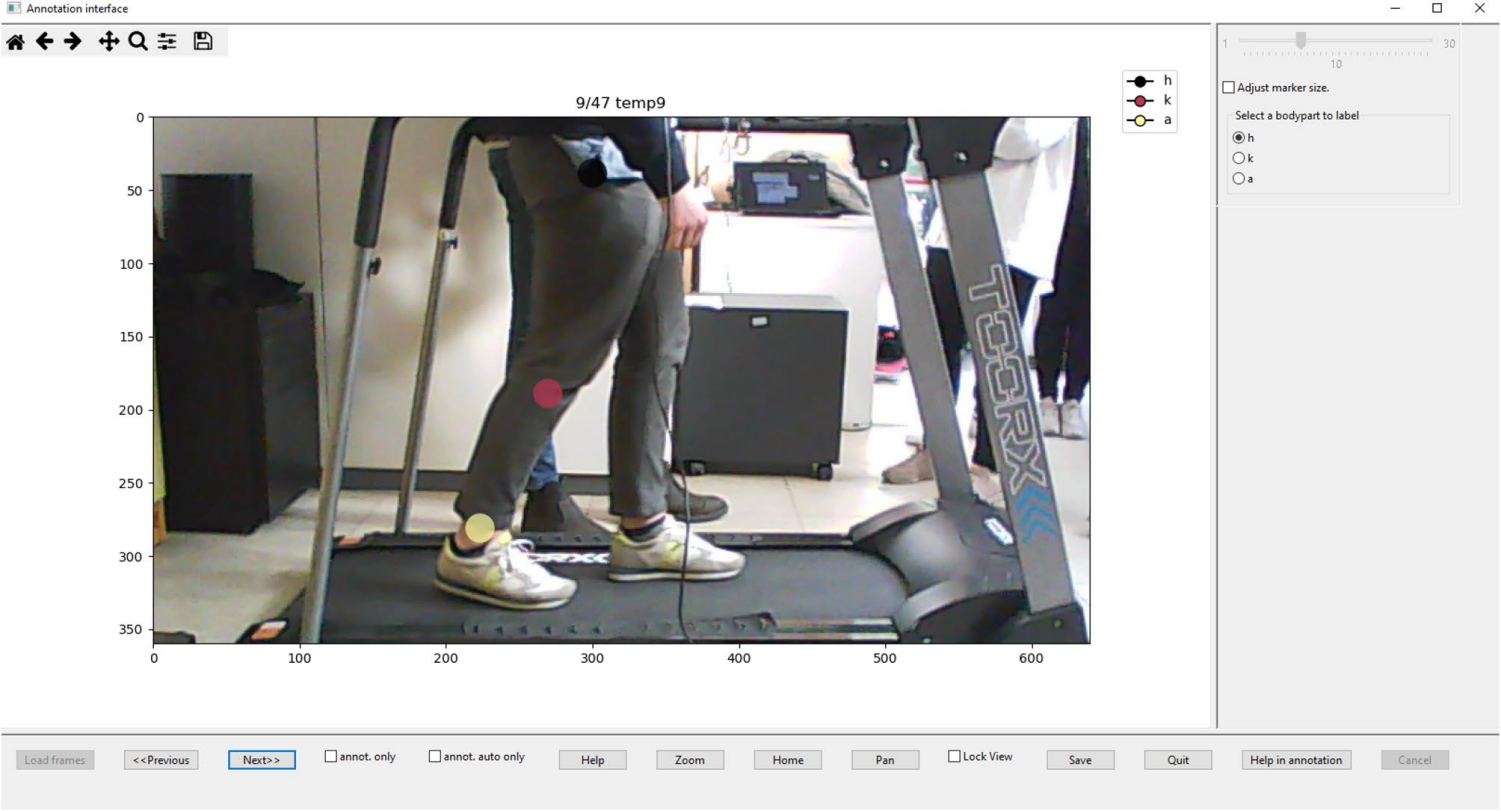
Example of VisionTool’s annotation GUI. The user can annotate keypoints of interest with the mouse, visualize images and the predictions overlaid on them.

In this paper, we present VisionTool, a Python toolbox for general-purpose markerless semantic features detection. VisionTool is based on transfer learning with deep neural networks, and has been designed to give appropriate importance to the following properties. (1) *Versatility*: the toolbox allows the user to define the semantic features to detect and to graphically annotate a set of training data to be used for further steps. (2) *Prediction accuracy:* precision in keypoints coordinates detection is a key factor in pose estimation since high-level features are later extracted from keypoints’ positions. (3) *Annotation efforts reduction*: after a minimal training set has been annotated (e.g., 5–25 frames, depending on the application domain), the toolbox offers the possibility to use an assisted annotation procedure. A neural network is trained on the annotated data, and used to predict the remaining frames (either the entire video or a random subset). The predictions are then automatically uploaded to the annotation tool and identified with different color maps with respect to the first set. The user can visually inspect the predictions, and correct mistakes dragging them with the mouse, adding or removing a label, in order to obtain a bigger annotated dataset, potentially improving further predictions. (4) *Simple and immediate adoption:* the toolbox is provided with an intuitive GUI that allows all the users to easily exploit all the implemented features (see Fig. 1). (5) *Modularity*: the toolbox is modular, meaning that new features and modules can be easily added to the package. (6) *Extensibility:* a key feature of VisionTool is the possibility to easily import a custom neural network model, integrating it with all the toolbox implemented features, using the available GUI. This feature supports longevity and usability of the toolbox, since it can be constantly updated with respect to the state-of-the-art architectures, as well as to exploit custom neural networks designed to solve specific problems. As shown in Section Results, VisionTool can be exploited in different ways. Firstly, it can be used as annotator, meaning that, given the frames composing one video or a set of images, a neural network can be trained on a subset of them and used to predict the remaining ones with high accuracy. In addition, the toolbox has good generalization properties (see “Generalization: prediction of unseen videos” section). Thus, it is possible to train a model on a set of frames belonging to one video and use it to detect the analogous set of selected keypoints in frames extracted from a different video.

With respect to state-of-the-art available toolboxes for semantic features extraction (e.g., DeepLabCut^24^) VisionTool presents the following main novelties: (1) The possibility to import and integrate a custom DNN model, extending the available set of fully convolution architectures and neural network backbones. (2) A higher number and variety of available pre-trained neural network models, as reported in the “Available deep neural network” section. (3i) The possibility to use the toolbox as assistant in the annotation process. In fact, a key-factor in VisionTool is the annotation GUI that allows to check and potentially correct an initial set of predictions, obtained with few training data, thus efficiently extending the annotations with minimal effort.

The reminder of the paper is organized as follows: first, we provide a schematic description of VisionTool’s algorithms, pipeline and GUI. Then, to test VisionTool’s versatility and precision, we apply the toolbox to three different domain of applications: (1) human action videos for action recognition; (2) face descriptors extraction and (3) plankton cell tracking. We show that, with less than 50 annotated frames, VisionTool is able to provide accurate features detectors ($mAP^{0.5} > 0.95$) for all the three included examples of application.

## Results

### VisionTool validation

**Figure 2:**
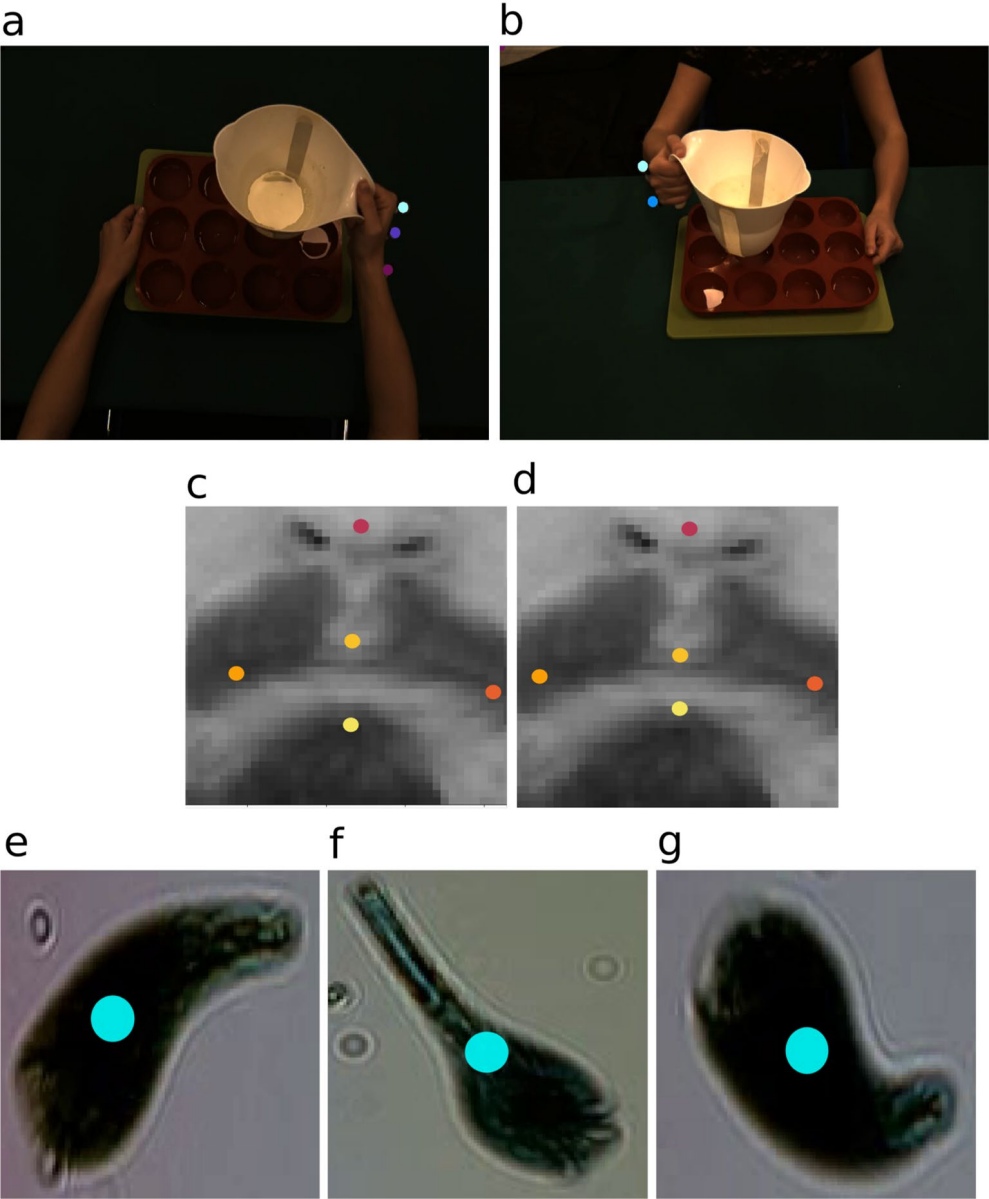
Examples of challenges for the datasets included in the work. (**a**, **b**) Moca’s keypoints occlusion in egocentric point of view (**a**) and frontal point of view (**b**) for action pouring-multi. It is common for such views to have little finger occluded by index, as well as wrist occluded by hand. (**c**, **d**) Mouth keypoints can be correctly annotated with a difference of several pixels. (**c**) ground-truth; (**d**) example of a different manual annotation. (**e**–**g**) stentor ceruleous contracting and relaxing during different stages of swimming with significant shape changing.

A semantic features detection toolbox should be versatile with respect to semantic, number of keypoints and domains of application, as well as precise, intuitive, and easy to use. To validate the VisionTool with respect to such requirements, we applied the toolbox to the analysis of three different datasets and associated application fields: (1) upper-body human actions from the Multiview Cooking Actions dataset (MOCA)^27^; (2) human faces from the Facial Keypoints Detection Kaggle’s dataset^28^; (3) videos of swimming plankton cells from the Plankton dataset^29^ (see Fig.  2). Each of them has specific challenges (reported in the following subsections) that support the evaluation of different aspects of the toolbox.

### Datasets

#### Multiview cooking actions dataset

The MOCA dataset^27^ collects video sequences acquired from multiple views of upper body actions in a cooking scenario. The purpose of MOCA is to provide a rich test bed to understand motion recognition skills and view-invariance properties of both biological and artificial perceptual systems. The dataset includes 20 cooking actions involving one or two arms of a volunteer and the tools to perform the correspondent action. Three different view-points have been considered for the acquisitions, i.e. lateral, egocentric, and frontal. Each action includes a training and a testing video, each containing, on average, 25 repetitions of the action itself. Since the dataset is multimodal, the volunteer was wearing markers in correspondence to the five keypoints considered for the detection task: (1) index; (2) little finger; (3) hand; (4) wrist and (5) elbow. However, no frames annotations are available with the dataset, so we needed to build a 2D ground truth for keypoints location to actually evaluate VisionTool’s features detectors accuracy. Hence, ground truth keypoints location was obtained exploiting VisionTool’s assistance annotation feature. The presence of physical markers makes the annotation process precise and repeatable, since it is immediate to build the annotation masks on top of the existent markers. On the other hand, occlusions and peculiar motion patterns represent a challenge for detecting the semantic features in the dataset (see Fig.  2a,b).

#### Facial keypoints detection dataset

The facial keypoints detection dataset^28^ was released for a kaggle competition focused on improving features detection accuracy in the context of face recognition. It contains $96\times 96$ pixels images of different subjects faces, with a total of 7049 training images and 1783 testing images. Complete annotations are only provided for a subset of training data. The detection task consists in identifying 15 facial keypoints, divided in 4 semantic groups: (1) eyebrow: left and right inner and outer limits; (2) eye: left and right eye center, inner and outer corners; (3) nose: nose tip, (4) mouth: left and right corners, top and bottom centers. Here, the challenge is mostly related to the low image resolution and the ambiguity in the identification (and annotation) of the keypoints (e.g., the top and bottom center of mouth, can be annotated and correctly predicted within a radius of several pixels, see Fig.  2c,d for an example).

#### Plankton dataset

The plankton dataset^29^ contains static images of swimming plankton extracted from 1-minute videos of 10 species of plankton acquired using a digital detector. The system used for acquisition employs the principles of a lensless microscope^30^. The dataset includes a total of 5000 images (500 per species) for training, and 1400 images for testing (140 per species). We evaluated VisionTool’s accuracy in detecting the center of the plankton cell. No ground truth was available, so we needed to annotate the data for actually evaluating VisionTool’s detectors accuracy. To perform annotation, first, we exploited an image-processing algorithm to select the centroid of the cell body (i.e., contour detection on available cell body masks, followed by selection of centroid for the contour with highest area). Then, we visually inspected the annotation with VisionTool’s annotation GUI, correcting the body cell center detection, when needed. In the plankton dataset, the challenge is represented by the low-resolution images and the intrinsic semantic of the keypoint to detect. For circular shape cells, in fact, it is trivial and precise the annotation process. However, few of the classes included in the dataset (i.e., the spirostomum ambiguum, the dileptus and the stentor coeruleous) can contract and relax (see Fig.  2e,f,g for an example), radically changing their shape, making hard and not unique the identification of the center cell for annotation and, consequently, for prediction.

### Evaluation metrics

VisionTool’s semantic features detection accuracy was evaluated in terms of mean Average Precision (mAP). As commonly done in literature and COCO challenges^31^, we computed mAP with respect to three different thresholds, defined as values of Object Keypoint Similarity (OKS): (1) 0.5; (2) 0.75; (3) average mAP value with OKS thresholds from 0.50 to 0.95 and steps of 0.05. Equation 1 reports the standard definition for OKS:

1 $$\begin{aligned} OKS = \sum _{i} e^{-d_i^2/2\sigma _i^2s^2}\delta (v_i>0)/\sum _{i}\delta (v_i>0) \end{aligned}$$

where $d_i$ is the distance between prediction and ground truth position for keypoint *i*,$\sigma _i$ is the per-keypoint standard deviation that controls fall-off, *s* is a scale factor and $v_i$ is a visibility flag. In our evaluation protocol, the standard deviation and the scale factor were computed with respect to keypoints mask area, and exploiting redundant annotations^31^. In our experiments, keypoints circle mask radius was set accordingly to the size of the semantic features to detect: 13 pixels for MOCA dataset (i.e., approximately the the size of the physical markers in the cooking videos); 2 pixels for faces, and 7 pixels for plankton dataset.

The notation mAP$^{0.5}$ corresponds to the mAP computed as in point (1); mAP$^{0.75}$ corresponds to the mAP computed as in point (2); while mAP refers to mAP computed as in point (3). In our evaluation metrics, the mAP at OKS $=$ 0.5 can be interpreted as the percentage of correct keypoints (PCK) metric^32^ (i.e., the fraction of predicted keypoints that fall within a threshold distance from the ground truth location) with a maximum allowed distance corresponding to an Intersection over Union (IoU) between ground truth and prediction keypoints masks equal to 0.5.

### VisionTool’s results on MOCA dataset

#### Automatic annotation accuracy

The toolbox can be adopted as an annotation assistant (i.e., trained on frames belonging to a certain video and tested on its remaining frames), to speed-up the annotation process while reducing user efforts. Annotation assistance is a key-feature in VisionTool, allowing to obtain a large set of high accuracy annotations with only few manually annotated frames. In fact, after few frames are annotated, the toolbox offers the possibility to train a neural network to provide coarse annotations and predict the remaining frames in the video. The prediction is then loaded in the same annotation interface used for the manual annotation, and potential mis-detected annotation points can be drag in the correct position (or missing labels added, if needed), to provide a final set of accurate annotations, that can be further used to train a more accurate model. We used the MOCA dataset testing videos for the three view points (i.e., lateral, egocentric and frontal) to validate VisionTool as automatic annotator, since no ground truth was provided with the dataset. The set of semantic features to detect includes: index, little finger, hand, wrist, and elbow. The first step consisted in using the toolbox to perform manual annotation of the 5 keypoints on a set of randomly extracted training frames. We used a random subset of 10 action videos among the 20 available from the lateral view-point to perform an automatic annotation accuracy evaluation as a function of the number of frames manually annotated. For this experiment, we used a LinkNet^33^ neural network with EfficientNetb1^34^ backbone pre-trained on ImageNet^35^ (RMSprop optimizer, weighted categorical cross-entropy as loss function, batch size equal to 5). The number of annotated frames was 10, 25 and 50. As expected, mAP increased with the number of annotated frames, reaching a maximum value of 0.974 for 50 annotated frames (see Table 1). A higher number of annotated frames could bring to higher detection accuracy, however, we limited our analysis to 50 frames, since the aim of the experiment is to test the toolbox potential with minimal manual annotation efforts.

**Table 1:** VisionTool’s detection accuracy with respect to number of annotated frames on MOCA dataset. A LinkNet with EfficientNetb1 backbone is trained on (i) 10; (ii) 25 and (iii) 50 frames, and used to predict the remaining ones, for each of the 10 lateral view point videos included in the evaluation subset. The results reported in this table correspond to the average mAP computed across the whole subset of videos.

# Frames	mAP$^{0.5}$	mAP$^{0.75}$	mAP$_{\text {index}}$	mAP$_{\text {little finger}}$	mAP$_{\text {hand}}$	mAP$_{\text {wrist}}$	mAP$_{\text {elbow}}$	mAP
10	0.888	0.869	0.818	0.855	0.866	0.882	0.890	0.862
25	0.979	0.966	0.852	0.862	0.967	0.888	0.890	0.892
50	0.984	0.977	0.949	0.972	0.975	0.986	0.989	**0.974**

Best results are in bold.

To show the importance of ImageNet fine-tuning, we trained and tested VisionTool’s semantic features extraction algorithms with randomly initialized weights. With the same number of annotated frames per video and the same neural network and backbone (i.e., LinkNet neural network with EfficientNetb1 backbone), keypoints predictions confidence is below the adopted minimum level of significance (i.e., 0.6 in our experiments), proving the importance of transfer learning to obtain high accuracy semantic features detectors with minimal training data.

As a next step, we evaluated the annotation accuracy with respect to the specific neural network applied. We used the same set of 50 annotated frames of previous step to compute prediction accuracy with 4 different neural networks (Unet^36^, LinkNet^33^, Pyramid Scene Parsing Network (PSPNet)^37^ and Feature Pyramid Network (FPN)^38^) and two popular neural network backbones in the computer vision literature: EfficientNetb1 and ResNet50^39^. As reported in Table 2, EfficientNetb1 outperformed ResNet50 for all the considered neural networks, with FPN and Unet leading to higher accuracy with respect to the other models. Table 3 provides information on the neural networks used in this experiment with respect to number of FLoating point Operations Per Second (FLOPs) and parameters. As we can see, even if Unet and FPN with EfficientNetb1 backbone accuracy are similar, the former works with a number of FLOPs significantly lower than the latter. Thus, having in mind the best compromise between efficiency and accuracy, we used Unet with EfficientNetb1 as backbone, and we trained it with 50 annotated frames to evaluate VisionTool’s annotation accuracy on the entire MOCA dataset. Table 4 summarizes the obtained results.

**Table 2:** VisionTool’s detection accuracy on MOCA dataset, with respect to neural networks and backbones. The 4 neural networks (i.e., FPN, LinkNet, PSPNet and Unet) are combined with EfficientNetb1 and ResNet50 backbone. Each model is trained on the 50 annotated frames, and used to predict the remaining ones, for each of the 10 lateral view-point videos included in the evaluation subset. The results reported in this table correspond to the average mAP computed across the whole subset of videos.

Net/Backbone	mAP$^{0.5}$	mAP$^{0.75}$	mAP$_{\text {index}}$	mAP$_{\text {little finger}}$	mAP$_{\text {hand}}$	mAP$_{\text {wrist}}$	mAP$_{\text {elbow}}$	mAP
FPN/Efficientb1	0.991	0.984	0.957	0.973	0.981	0.987	0.991	**0.978**
FPN/ResNet50	0.975	0.944	0.874	0.949	0.923	0.978	0.985	0.942
LinkNet/Efficientb1	0.992	**0.987**	0.974	0.945	0.985	0.971	0.976	0.969
LinkNet/ResNet50	0.858	0.849	0.769	0.786	0.859	0.788	0.890	0.819
PSPNet/Efficientb1	0.987	0.957	0.894	0.929	0.928	0.957	0.949	0.931
PSPNet/ResNet50	0.983	0.914	0.803	0.867	0.850	0.927	0.935	0.876
Unet/Efficientb1	**0.993**	0.981	0.962	0.976	0.978	0.984	0.976	0.970
Unet/ResNet50	0.952	0.945	0.848	0.875	0.878	0.887	0.975	0.893

Best results are in bold.

**Table 3:** Neural networks and backbones complexity in terms of FLoating point Operations Per Second (FLOPS), number of parameters and layers.

Net/Backbone	FLOPS (bilions)	# Params (milions)	# Layers
FPN/Efficientb1	12.80	0.96	379
FPN/ResNet50	6.43	2.69	237
LinkNet/Efficientb1	8.04	0.86	388
LinkNet/ResNet50	2.09	2.88	246
PSPNet/Efficientb1	1.76	0.18	142
PSPNet/ResNet50	0.90	0.39	116
Unet/Efficientb1	8.72	1.26	373
Unet/ResNet50	2.58	3.26	231

**Table 4:** VisionTool’s detection accuracy on MOCA dataset, when used as annotator. A Unet with EfficientNetb1 backbone is trained on 50 frames, and used to predict the remaining ones, for each of the 60 videos included in the dataset. The results reported in this table correspond to the average mAP computed across the whole set of videos.

View point	mAP$^{0.5}$	mAP$^{0.75}$	mAP$_{\text {index}}$	mAP$_{\text {little finger}}$	mAP$_{\text {hand}}$	mAP$_{\text {wrist}}$	mAP$_{\text {elbow}}$	mAP
All together	0.992	0.987	0.974	0.945	0.985	0.971	0.976	0.970

#### Generalization: prediction of unseen videos

We showed that VisionTool is able to provide high-accuracy semantic features detectors with minimal annotated data, when used as annotator (i.e., trained on frames belonging to a certain video and tested on its remaining frames). However, when dealing with semantic features extraction tasks, generalization properties are crucial, since the same keypoints will have to be accurately detected in different testing videos with respect to the training ones. This is especially true in pose estimation tasks, where different subjects performs the same action in different environments. To investigate how the algorithms implemented in the toolbox generalize and perform on unseen videos, we used each view-points set of 20 videos to perform a k-fold experiment, with k $=$ 5, each time using onefold as testing data and the remaining four to train the detection algorithms (16 training and 4 testing videos per fold). As we can see in Table 5 the toolbox is able to provide features detectors that generalize well between different videos. In fact, the mAP$^{0.5}$ is higher than 0.95 for all of the considered set of videos, while the mean mAP across the 5 different folds, is higher than 0.90. As expected, the elbow and the wrist are the easiest keypoints to detect, since they are the most stable with respect to different videos, while the index and the little-finger are the hardest ones, since they are the most variable and the ones characterized by the highest level of motion. Finally, as expected, the frontal view-point is the hardest one to predict, since videos acquired with such view-point present the highest variability of keypoints detection and number of occlusion with respect to the 20 cooking actions. As a final step, we investigated how accurate are VisionTool’s detections when trained on three different view-points videos at once. Hence, we trained a neural network on the entire dataset with a k-fold approach (k $=$ 5). We split the dataset into the fivefolds imposing to have the same number of videos belonging to the three different view-points at each fold (i.e., 16 videos per each view for training and 4 videos for testing, for a total of 48 training and 12 testing videos per fold) obtaining a corresponding mAP equal to 0.908.

**Table 5:** VisionTool’s detection accuracy on MOCA dataset. A k-fold (k $=$ 5) approach is used for each view point (i.e., the detectors are trained on fourfolds and the remaining one was predicted). The results reported in the table correspond to the average mAP computed across the different folds.

View point	mAP$^{0.5}$	mAP$^{0.75}$	mAP$_{\text {index}}$	mAP$_{\text {little finger}}$	mAP$_{\text {hand}}$	mAP$_{\text {wrist}}$	mAP$_{\text {elbow}}$	mAP
Lateral	0.969	0.905	0.865	0.845	0.889	0.958	0.988	0.909
Egocentric	0.962	0.929	0.925	0.789	0.963	0.922	0.978	0.915
Frontal	0.957	0.858	0.861	0.907	0.836	0.930	0.992	0.905
All together	0.954	0.904	0.880	0.821	0.912	0.949	0.980	0.908

### Face dataset results

In this section, we evaluated if VisionTool is able to provide accurate features detection for the face dataset. We extracted a set of 1500 images from the training set provided with full annotation. We split the dataset in training and testing with ratio 3:1, resulting in 1000 images for training and 500 for testing. We evaluated the 4 neural networks included in VisionTool (i.e., Unet, LinkNet, PSPNet and FPN) with EfficientNetb1 backbone, (considering that on the MOCA dataset this was the best performing backbone, batch size equal to 5, RMSprop optimizer). Table 6 summarizes the obtained results in terms of mAP. The detector based on FPN and EfficientNetb1 shows the highest detection accuracy, with a mAP$^{0.75}$ around 0.96 and a mAP of 0.86.

**Table 6:** Facial keypoints detection accuracy in terms of mAP. EfficientNetb1 is used as backbone for the 4 neural networks implemented in VisionTool. The 15 detected Keypoints are divided into 4 semantic groups, as explained in “Facial keypoints detection dataset” section.

Net/Backbone	mAP$^{0.5}$	mAP$^{0.75}$	mAP$_{\text {eyebrow}}$	mAP$_{\text {eye}}$	mAP$_{\text {nose}}$	mAP$_{\text {mouth}}$	mAP
FPN/Efficientb1	0.998	0.958	0.791	0.939	0.739	0.926	**0.859**
LinkNet/Efficientb1	0.998	0.950	0.771	0.920	0.724	0.908	0.838
PSPNet/Efficientb1	0.992	0.896	0.742	0.915	0.636	0.878	0.803
Unet/Efficientb1	0.994	0.934	0.749	0.905	0.708	0.896	0.824

Best results are in bold.

### Plankton dataset results

As a final quantitative application, we evaluated if VisionTool is able to provide an accurate detector for the center of the plantkon cell body. We considered the testing set of 140 images for each of the 10 included classes of plankton in the dataset, for a total of 1400 images. We considered only the testing set because it contains a sufficient number of images to accomplish our task and because in this way we reduced labeling efforts. For each class, we annotated a random set of 50 images, as previously explained. After ground truth annotations have been created, we trained the 4 neural networks included in VisionTool with the same configuration adopted for the Face dataset (previous subsection) on the 50 images for each class, and predicted the plankton cell center on the 90 remaining images. Table 7 shows the performances in terms of mAP. Despite the intrinsic morphology change and the arbitrarity in the keypoint annotation, VisionTool was able to achieve high detection accuracy. The most accurate detectors correspond to a FPN and Unet with EfficientNetb1 backbone, reaching a mAP$^{0.75}$ averaged across the 10 classes equal to 0.92 and a mAP around 0.91.

**Table 7:** Plankton cell center detection accuracy in terms of mAP. EfficientNetb1 is used as backbone for the 4 neural networks implemented in VisionTool.

Net/Backbone	mAP$^{0.5}$	mAP$^{0.75}$	mAP
FPN/Efficientb1	0.980	0.919	**0.908**
LinkNet/Efficientb1	0.951	0.837	0.839
PSPNet/Efficientb1	0.942	0.776	0.784
Unet/Efficientb1	0.976	0.919	0.907

Best results are in bold.

## Discussion

This paper introduces VisionTool, a toolbox for semantic features extraction. To facilitate broad usage and scientific community contribution, the toolbox and a detailed user guide are available at https://github.com/Malga-Vision/VisionTool.git. We showed that transfer-learning from pre-trained deep neural network can be quickly applied to completely different contexts and applications (from cooking actions to swimming cells) with accurate results. We believe that VisionTool could supplement the list of available toolboxes for video analysis, allowing even inexperienced users to obtain high-accuracy features detectors for a wide range of applications.

### Dataset annotation and performances

VisionTool is based on transfer-learning from ImageNet pre-trained deep neural networks. Transfer-learning combined with the implemented training strategies, that include data augmentation, the possibility to easily customize the model hyperparameters (e.g., the optimizer, the learning rate, the number of epochs, the batch size and the loss function), the availability of a weighted version of the loss functions with a customized weights computation to handle class imbalance and the implementation of basic learning strategies (i.e., learning rate scheduling and early stopping) allow to obtain high-accuracy detectors with minimal annotated training data. In our experiments, we showed that 50 frames were sufficient to obtain high accuracy detectors ($mAP>0.9$) for the three investigated datasets. In general, the accuracy of fine-tuned features detectors may depend on the number and quality of annotations. A precise labeled training set may be not trivial to obtain, it is time-consuming and user-dependent. As a solution, our toolbox offers the possibility to obtain an additional set of data with an automatic procedure, where a deep neural network is trained to predict a subset of frames, with predictions that are later available in the annotation GUI for checking and potential correction. We used such procedure to obtain a ground truth for the MOCA dataset, where annotations were not provided with data. However, in noisy videos where objects move with high frequency, frames where this particular behavior is present could be not part of the randomly selected minimal annotated set for training. The exclusion of such frames from training potentially brings to sub-optimal results. In such cases, a solution comes directly from VisionTool’s output, with a post-processing training frame addition. In fact, VisionTool provides as output confidence maps (of the same size of the input image) for each keypoint, where pixel intensity corresponds to the confidence of that pixel belonging to the detected keypoint. These maps (also called probability maps) are thresholded with a minimum level of confidence to provide the final predicted keypoints locations. Hence, frames with particularly low level of confidence could be added to the training set to test if the accuracy can be improved. Low values in the probability maps could also occur when keypoints are occluded. In this case, multiple view-points (as in the MOCA dataset) are ideal to improve precision in features extraction.

### VisionTool’s versatility

We showed that VisionTool is able to provide accurate detections for three different datasets: (1) MOCA; (2) facial keypoints detection and (3) swimming plankton cells. We chose such datasets because their different features supported the evaluation of specific aspects of the proposed toolbox. In the MOCA dataset, in fact, even if videos were acquired by three different view-points, it was still possible to obtain high-accuracy (mAP > 0.9) when detectors were trained with different view-points videos at once. In the face dataset, we showed that VisionTool provides accurate detectors (mAP > 0.9) when input data are sequences of static low-resolution images and features are smaller and more user-dependent with respect to previous dataset (where annotations coincide with physical marker positions). Finally, the plankton dataset has low-resolution images and the position of cell centroid is ambiguous and strongly dependent from the user. To prove this point, we asked three different annotators to provide annotations for 50 frames per each class. The standard deviation among the different set of annotations reached a maximum value of 7 pixels for the class dileptus, where strong intrinsic morphology change and the shape of the cell make harder to precisely identify its centroid. However, VisionTool’s was still able to train an accurate detector (mAP > 0.9) for each of the ten species of plankton included in the dataset.

### VisionTool’s computational cost details

The deep neural network embedded in the toolbox were trained and tested on resized version of the original video frames (in the current version, to size $288\times 288$), that were later scaled to the original size with no effect on features detection accuracy. Thus, VisionTool’s semantic features extraction can be quite fast on modern hardware. For instance, inference rate for the MOCA dataset spanned from 50 to 85 Hz on a Nvidia RTX2060 with 6 GB of RAM (for Unet with EfficientNetb1 backbone). Such prediction time makes VisionTool compatible with real-time features detection applications. The inference time could be further decreased by increasing the resize rate, cropping the frames, or modifying the architectures (e.g., with pruning algorithms) to speed up the prediction process.

### VisionTool’s extensibility

VisionTool includes four largely used fully convolutional architectures for detection and segmentation, with 30 different ImageNet pre-trained neural network models to be used as backbones. Such architectures, together with the implemented training strategies, generally allow to obtain accurate detectors with minimal annotated training data. However, a toolbox for general-purpose markerless semantic features detection should ideally be extensible, allowing the user to exploit all the toolbox implemented features with custom neural network models. Such extensibility is fundamental for two main reasons: (1) longevity and usability: the possibility to import custom architectures may be fundamental to keep the toolbox updated with the state-of-the-art as well as to exploit neural networks appositely designed for the solution of a certain problem or tailored to certain properties of an investigated dataset; (2) two-stage fine tuning: the possibility to easily upload a neural network model pre-trained on a certain custom dataset, and re-use the information stored in its weights to perform fine-tuning on a different problem, may be useful and lead to accuracy improvement. For these reasons, extensibility is a key-point of novelty in VisionTool with respect to the state-of-the-art semantic features extraction toolboxes. In particular, it is possible to upload and integrate a custom deep neural network model, including a pre-trained custom model on a certain dataset, in order to re-use the learnt weights, still exploiting all VisionTool implemented functionalities, including the annotation interface, the training strategies, data augmentation, prediction visualization and corrections. To improve user experience, a custom model can be simply imported using an apposite button in the corresponding GUI.

## Methods

In this section we formulate the machine learning problem underlying semantic feature detection, we provide a schematic description of VisionTool’s features extraction pipeline and give a detailed report on the available implemented neural networks.

### Machine learning problem formulation

VisionTool’s semantic features detection algorithm is structured as an image segmentation task, in the form of a multi-class classification problem. More formally, let us represent a dataset as a set of N images $I = \{I_0,I_1,..,I_N\}$ with pixels $\pmb {x}(x_1,x_2)$ on a discrete grid $m \times n$ with intensities $\pmb {I_i}(\pmb {x}) \in \, J$ $\subset R$. Let us split the dataset *I* into three separated subsets: $I_{TRAIN}$ for training, $I_{VAL}$ for validation and $I_{TEST}$ for testing. For each training (and validation) image $I_i$ we assume a ground truth is available as a set of binary segmentation masks $M_{Il}$ with pixels intensities $\in$ [0,1]; $l \in [0,L]$ represents the semantic label, and *L* is the number of keypoints to detect. Let $M'_I$ be the cumulative ground truth matrix, with pixel intensities $\in [0,L]$. A multi-class neural network is trained to learn a function $F:I \xrightarrow {} M'$ that maps each pixel **x** $\in I$ to its semantic label *l* with some probability. To maximize such probability, a loss function is defined to estimate the deviation of the network prediction from ground truth, at each training step (i.e., the training error). To minimize the prediction error, the loss function is decreased iteratively with training, until a defined set of stopping criteria is met. Since we are only interested in detecting a set of defined keypoints, a background class is added to the set of semantic labels. Thus, each pixel of an image can be assigned either to one of the keypoints classes or to the background. Considering that the pixels belonging to the keypoints area are generally significantly less than the ones belonging to the background (i.e., everything in the image which is not a keypoint to detect), the problem becomes an imbalanced multi-class classification problem, and imbalance between classes is handled by using a set of weights for each class, with an inverse proportion with respect to the number of pixels belonging to the specific feature class. Hence, we adopted a weighted version for the implemented loss functions and a customized approach to set the correspondent weights, depending on number of keypoints and background pixels.

### VisionTool’s workflow

**Figure 3:**
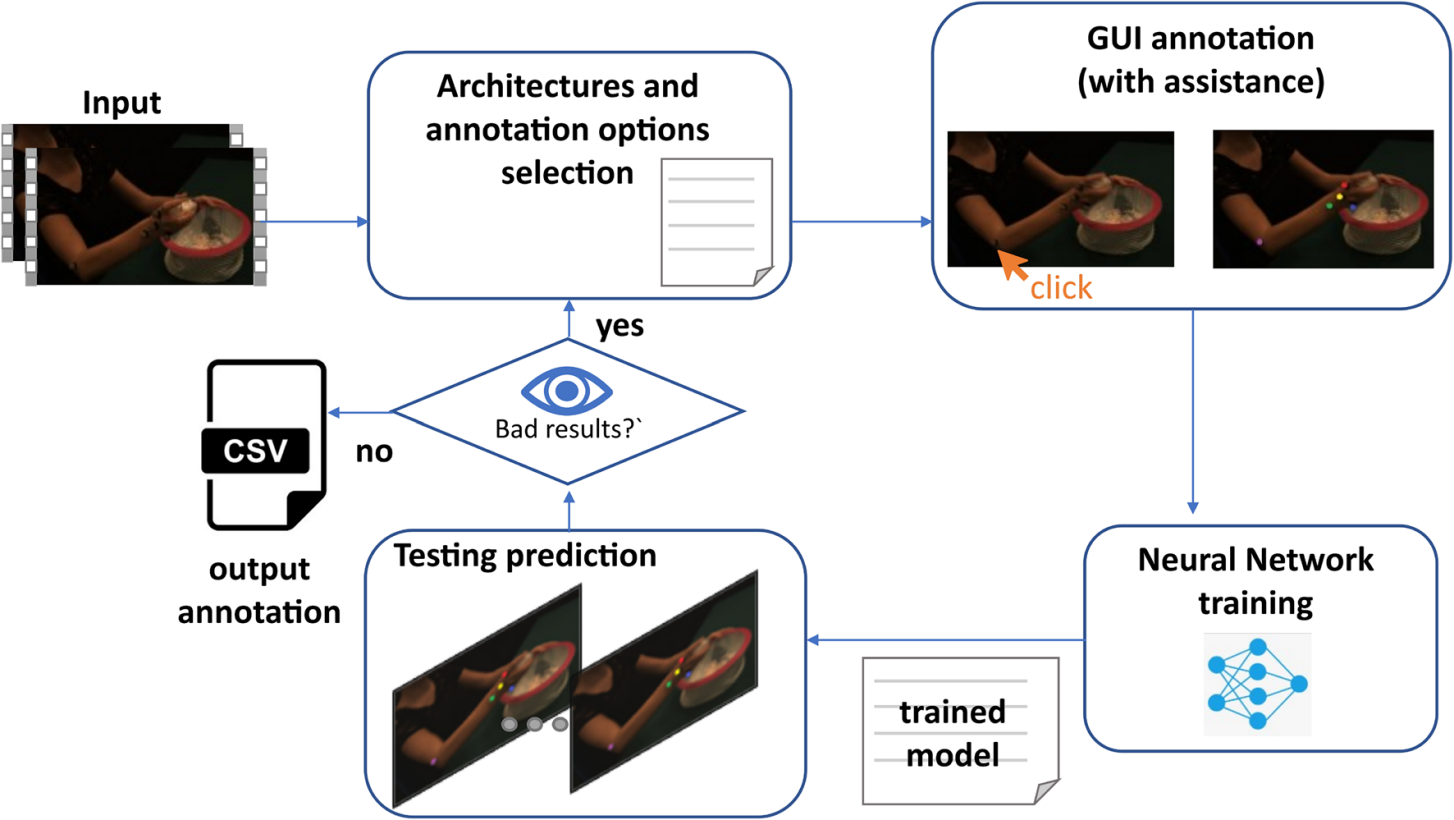
VisionTool’s workflow description.

VisionTool is a semantic features extraction toolbox written in Python based on tensorflow and the embedded neural network library keras (see Fig. 3 for a schematic description of VisionTool’s workflow). The toolbox offers a user-friendly interface allowing the user to easily exploit all the implemented features. First, the user creates a new project, imports input data (videos or set of images), defines the keypoints (i.e., the semantic features to detect) and selects the number of frames to be annotated, which is randomly extracted from the total available set. The number of frames to be annotated (i.e., the training set size) is a fundamental parameter for the features detection task. A meaningful choice should be a compromise between annotation efforts and the quality of prediction. In general, it depends on the difficulty of the specific task (e.g., number of keypoints, percentage of occlusions, average standard deviation of keypoints location, number of poses in pose estimation applications). To manually perform features annotation, the user exploits the dedicated annotation interface (see Fig. 1), using the mouse to select the keypoints (e.g., keypoints coordinates in human-pose estimation). A deep neural network is chosen among the available ones and trained on the annotations. Data augmentation based on random transformations (i.e. rotation, shearing, zooming and shifting) is performed at training time to allow for better generalization ensuring high accuracy on few training data. The trained model is then ready to be used to perform features extraction in testing videos (either unseen videos, or the remaining frames of the training video). After testing, the obtained results can be visually inspected by the user; if they are not satisfactory, they can be corrected and used as a further set of annotated data in the training procedure, implementing an active learning framework^40^. VisionTool’s GUI guides the user through the entire process of semantic features extraction. More details on the main steps are reported in the next subsections.

### Input data import and annotation

After a project is created (or an existing project is opened), the user can add new videos (or process the existing ones). The videos are automatically read by the toolbox to provide the total length (in number of frames), helping the user to set a valid number of frames to annotate. After the user sets the number *j* of frames to annotate, a random set of *j* frames is extracted among all the available ones and annotated.

When the user annotates an image, a circle with radius *r* is draw over the frame in the annotation tool, where *r* can be set by the user through the annotations option GUI. Such circles are then used to form the ground truth segmentation masks.

### Vision Tool as an annotation assistance tool

The larger the training set, the higher the algorithm precision in detecting the semantic features from videos. However, the annotation procedure is time consuming, forcing to choose a compromise between number of annotations and prediction accuracy. In order to partially solve this issue, VisionTool implements a deep neural network-based automatic annotation procedure. After at least 10 frames are manually annotated, in fact, there is an option to train a deep neural network, to provide an initial annotation estimation for a number *k* of randomly extracted frames, with *k* input by the user. After the prediction, the automatic annotated frames are loaded in the same GUI used for manual annotation, and the user can check the results and correct potentially mis-predictions by dragging the points to the correct location, adding or removing a detected keypoint, with a significant saving in term of annotation efforts. The automatic and manual frames predictions are represented with different color maps in order to be clearly distinguishable in the GUI. The checked and corrected frames are added to the original set of manual annotations to increase the training set size. The automatic annotation tool is a key feature and the main novelty in VisionTool, reducing the user annotation efforts while speeding up the entire features detection process, eventually leading to a higher prediction accuracy and better generalization.

### Available deep neural networks

VisionTool includes 4 different largely used architectures for detection and segmentation: UNet^36^, LinkNet^33^, Pyramid Scene Parsing Network (PSPNet)^37^ and Feature Pyramid Network (FPN)^38^. These architectures encode the input exploiting sequential downsamples (i.e., compressing the images) and then reconstruct the input by specular sequential upsamples with different combinations with respect to the downsamples layers according to the specific architectures. The encoding module can be adapted from different neural networks, choosing the number of parameters and network depth according to the specific pose estimation problem. VisionTool offers 30 models (including EfficientNets, ResNets and MobileNets) to be used as backbones for each of the available deep network. A key-feature in VisionTool is the possibility to obtain high accuracy in the semantic features extraction with a limited training set (i.e., with limited annotations). Such feature is implemented exploiting transfer learning, providing better generalization than training from scratch. In fact, ImageNet pre-trained weights are available for each of the neural network backbones. Neural networks implementations is based on the library proposed in^41^.

### Model training

A dedicated GUI offers the possibility to select the neural network, the optimizer, the loss function, the learning rate and the number of epochs to wait if validation loss does not decrease before stopping training, training from scratch or using transfer-learning from ImageNet pre-trained weights. The learning rate is halved at every *z* epochs to facilitate the convergence of the trained model, and *z* is again set through the dedicated architectures preferences GUI. Data augmentation with random rotation, distortions, zooming and shifting is performed during training to improve model generalization. The training is performed with a mini-batch approach, with batch size set by the user with the dedicated GUI. In the current version, the two available loss functions are (1) categorical cross-entropy and (2) dice loss. Both the loss functions are adopted in a weighted version, with weights defined as described in the “Machine learning problem formulation” section.

### Model deployment

After training, VisionTool can be used to annotate other frames of the same videos or new videos. The final locations of the detected keypoints are obtained by thresholding the confidence maps. Confidence maps (one per keypoint in each image) have pixels intensities corresponding to the probability of finding the keypoint at that precise location (the higher the intensity, the higher the algorithm confidence about the pixel belonging to that specific keypoint). A hard threshold is applied to the predicted image (the threshold is set by the user through the architectures preferences GUI). Such threshold corresponds to a tolerance, as the minimum value of accepted confidence for a prediction. The final estimation confidence is computed as the average grey level value of the thresholded predicted masks, while the corresponding centroid is used as keypoint’s estimated location. VisionTool’s final output corresponds to a dataframe reporting the estimated locations for the detected features in each frame, stored both as a h5 and a csv file. They include the detected keypoints coordinates and the corresponding estimation confidence for each of the video frames. If one of the keypoints has not been detected in a certain frame, the corresponding output coordinates are automatically set to a negative number (i.e., the point ($-1, -1$)). The toolbox offers the possibility to save the predicted maps for each keypoint for user visual inspection or further processing.

### Ethics declarations

The MOCA dataset is publicly available and described in^27^. The kaggle face recognition dataset is publicly available and accessible at^28^. For the ethics declaration we refer to the original datasets publication.

## Data Availability

All the datasets analyzed during the current study are publicly available. The MOCA dataset is publicly available at https://sites.google.com/view/themocaproject/welcome and described in^27^. The kaggle face recognition dataset is publicly available and described at https://www.kaggle.com/c/facial-keypoints-detection/data^28^. The lensless dataset of freshwater plankton is publicly available at https://www.dropbox.com/s/kb96xzmxlrfii3k/LENSLESS%20DATASET.zip?dl=0 and described in^29^.
